# Assessment of a Parent-Child Interaction Intervention for Language Development in Children

**DOI:** 10.1001/jamanetworkopen.2019.5738

**Published:** 2019-06-14

**Authors:** Dimitri A. Christakis, Sarah J. Lowry, Georgia Goldberg, Heather Violette, Michelle M. Garrison

**Affiliations:** 1 Department of Pediatrics, University of Washington, Seattle; 2 Seattle Children’s Research Institute, Seattle, Washington; 3 Department of Health Services, University of Washington, Seattle

## Abstract

**Importance:**

Parent-child interactions are critical for language development in early life.

**Objective:**

To test whether a clinic-based intervention was associated with improved home language environment and language development.

**Design, Setting, and Participants:**

A pre-post study of a multifaceted clinic-based intervention called Talk It Up was conducted for 61 English- or Spanish-speaking families with children aged 2 to 12 months. Enrollment took place at a single urban pediatric clinic from January to November 2017, with follow-up data collection through May 2018. Analyses took place from June to August 2018.

**Interventions:**

The 6-month multifaceted intervention consisted of delivering Language Environment Analysis word counts with clinician feedback, coaching, and twice-weekly pushes of brief instructional videos via a commercially available smartphone application.

**Main Outcomes and Measures:**

Baseline and follow-up *z* scores for adult word counts, parent-child conversational turns, and child vocalizations and language development as measured by the Developmental Snapshot score were compared and adjusted for confounders.

**Results:**

Among 61 families, the mean (SD) child’s age at baseline was 5.9 (3.3) months. English was the primary language spoken in the home for 54 families (89%). At follow-up, Talk It Up was associated with significant improvements in adult word counts (mean *z* score, 0.30; 95% CI, 0.05-0.55), parent-child conversational turns (mean *z* score, 0.29; 95% CI, 0.002-0.59), and Developmental Snapshot score (mean, 6.59; 95% CI, 0.95-12.23), but there were no improvements in child vocalizations (mean *z* score, −0.13; 95% CI, −0.49 to 0.24).

**Conclusions and Relevance:**

A multifaceted clinic-based approach to promote parent-child interactions holds some promise. Future, larger studies are warranted.

## Key Points

**Question:**

Was a clinic-based multimodel intervention, including a smartphone app and coaching, associated with improved language environment for children 2 to 12 months of age?

**Findings:**

A pre-post study was conducted for 61 families with children aged 2 to 12 months. The intervention was associated with significant improvements in the Developmental Snapshot score and mean *z* scores for adult word count and parent-child conversational turns, but there were no improvements in mean *z* score for child vocalization count.

**Meaning:**

A multifaceted clinic-based approach to promote parent-child interactions holds some promise, but larger studies are warranted to assess effectiveness and broader generalizability.

## Teaser

This pre-post study evaluates whether the Talk It Up intervention for parents of children aged 2 to 12 months, which uses direct coaching and a smartphone app, is associated with improvements in adult word counts, parent-child conversational turns, child vocalization counts, and language development.

## Introduction

In their seminal work, Hart and Risley^1^ found that low-income children grow up in a considerably impoverished linguistic environment. Specifically, by the time they start kindergarten, low-income children hear approximately 30 million fewer words than do their high-income counterparts. This disparity has been confirmed multiple times.^2,3^ However, the word gap is only part of the problem. Equally important is the paucity of reciprocal exchanges between caregivers and children, called *conversational turns*, which help build social, cognitive, and emotional capacity.^4^ Recent data suggest that diminished parental engagement is becoming a problem for all children regardless of class. Distracted parenting, whereby parents are looking at their devices instead of interacting with their children, is a phenomenon that does not discriminate on the basis of socioeconomic status.^5,6,7^ Accordingly, interventions are needed to help promote parent-child interactions and to foster reciprocal vocalizations across all income strata. Primary care sites have been previously used for interventions to promote child-parent interactions through in-person teaching and coaching using videos and book distribution.^8,9,10,11^ Although such interventions are effective, they are also labor intensive and require considerable in-person counseling.

Given that parental device use may be part of the problem, we were curious how it might be leveraged to become part of the solution. Specifically, if parents are using their phones when with their children, can their phones be used to deliver timely child development tips that promote interactions rather than distraction? Can we build on prior clinic-based work to combine some in-person coaching with contemporaneous video delivery that would occur in real-world settings? We therefore designed and tested a multifaceted intervention designed to enrich the linguistic environment of children younger than 18 months.

## Methods

### Setting

We devised Talk it Up as a multicomponent program to be disseminated through primary care offices. The goal was to leverage the fact that children aged 2 to 18 months have, per American Academy of Pediatrics guidelines, 7 recommended well-child care visits (WCVs). We sought to make language the fifth vital sign and to encourage pediatricians to emphasize it as part of their examinations. The study design and intention were communicated to all clinicians during a 30-minute presentation. They were informed that study patients’ medical records would now include a language environment assessment including the child’s language environment percentile. Clinicians were free to do whatever they wanted with that information, but we encouraged them to use it to emphasize the importance of talking to one’s child. We conducted this pilot study in the Odessa Brown Children’s Clinic, which is affiliated with Seattle Children’s Hospital. Located in the Central District of Seattle, Washington, Odessa Brown serves a diverse patient population including both low- and middle-income families. The Seattle Children’s institutional review board approved the study protocol. Written informed consent was obtained from all parents or guardians. Enrollment was open from January 2017 to November 2017.

### Intervention

The intervention was designed to provide short actionable tips and feedback to promote behavioral change. It has several components: (1) the Language Environment Analysis (LENA) system, (2) clinician (physician and advanced registered nurse practitioner) feedback, (3) the ReadyRosie smartphone application (app), and (4) coaching. The LENA system is a digital language processor that young children wear in a customized vest; it records everything a child hears and says, as well as conversational turns. A proprietary software system decodes the recordings, generating adult words, child vocalizations, and conversational turns over time, and provides parent-friendly reports as well as quantitative data for research. The LENA system has been used extensively in research, and further details can be found elsewhere.^12,13^

The LENA system was used both to collect outcome data and to present parents and their medical clinicians with a current state of their children’s linguistic milieu. The LENA reports of the number of words were appended to each patient’s medical records at scheduled WCVs. These reports, akin to growth curves, plot the number of words a child hears compared with age-specific norms to provide a percentile. The LENA reports were explained to clinicians before the study launch. The reports were appended to patients’ visit documentation and handed to clinicians but not embedded in the medical record. The expectation of clinicians was only that they would use the records to encourage additional talking or to reinforce what was currently being achieved. The LENA data were collected at 2 points: baseline (time 1) and a follow-up visit (time 2) that took place before a subsequent scheduled WCV appointment, whenever they occurred.

The LENA devices and vests were sent to families at each point with a prepaid envelope to return the hardware. The LENA data were automatically uploaded to the cloud and made available to the research team. At each point, children wore the LENA vests for 2 days during a single week, for 16 hours each day.

Once baseline data were collected, research assistants met families in the waiting room after their clinician visit. They reviewed the baseline data, provided coaching about times during the child’s day when interactions were minimal according to the LENA data, and registered participants to receive the app’s message delivery via text message or email once the app was installed on participants’ smartphones. They further instructed parents that the app would deliver practical ways to promote conversation. Each coaching session lasted less than 15 minutes.

The app delivers links to short (30-60 seconds) age-specific videos to users’ smartphones. These videos use real families to model ways to verbally interact with one’s child during routine activities (eg, diaper changes, shopping, and cooking). The frequency with which these are sent is customizable. We opted to deliver 2 messages per week and arranged for them to be age based. Videos were arranged in age bands (eg, 2-5 months, 5-8 months, and so forth) and delivered sequentially. Users can opt to click on the link (or not), and we were able to collect data on whether they did so.

Families had a minimum of 30 days of access to the app before being asked to do a LENA follow-up appointment. After 30 days, they were scheduled to do the follow-up recordings by mail as soon as possible, but before their next scheduled WCV appointment. Because this was a pragmatic effectiveness trial, the number of weeks before the scheduled care visit varied and was dependent on parents scheduling a follow-up WCV. At the next WCV, LENA data were made available to their child’s clinician at the visit and were reviewed with a research assistant afterward for additional coaching.

### Participants

All developmentally normal children between the ages of 2 and 12 months at enrollment were eligible for participation. Potentially eligible families were identified by upcoming clinic visits in the clinic’s electronic scheduling system. Families for whom neither Spanish nor English was the predominant language spoken at home were not eligible because the app currently is not available in other languages.

Parents completed a baseline demographic questionnaire and baseline LENA assessment at enrollment. Owning a smartphone was a requirement for eligibility. Participants received $20 gift cards for returning the baseline survey and each of the LENA assessments.

### Design

We used a pre-post design, with a baseline and 1 follow-up assessment. Although a randomized clinical trial design would have allowed stronger causal inference, it was the expressed preference of the clinic administration that all families be offered the intervention.

### Outcomes

We analyzed the following outcomes, all of which are mean hourly counts of an expressive language construct as detected and coded by the LENA system: adult word count (AWC), representing the mean number of total words spoken by adults per hour within the child’s hearing; child vocalization count (CVC), representing the mean number of distinct words, babbles, or prespeech vocalizations by the child per hour; and parent-child conversational turns (PCCTs), representing the number of language rallies between the child and any adult, for which a vocalization by the child and a response from the adult, or vice versa, is counted as a single conversational turn. Because each of these constructs would be expected to increase in number over time even in the absence of an intervention, the software provides these outcomes as both raw counts and transformed as *z* scores for age in months; these analyses use the latter. The *z* scores can be interpreted as standardized effect sizes, or standard deviations from the mean of the population norms for that age in months; as a result, differences in *z* scores can be interpreted as standardized effect sizes, as measured with Cohen *d* values. In addition, we collected data using the Developmental Snapshot, a 52-item measure of parent-reported language skills for infants and toddlers. The Developmental Snapshot has many advantages over existing measures in that it is short (<15 minutes), parent reported, and sensitive to subtle changes. The Developmental Snapshot has shown excellent validity compared with both the Preschool Language Scale, Fourth Edition and the Receptive-Expressive Emergent Language Test, Third Edition (correlation coefficient, 0.93-0.96).^14^ All outcomes were ascertained at both baseline and follow-up.

### Statistical Analysis

Baseline parent and infant demographic data and other participant characteristics were examined graphically and summarized descriptively. Because nonlinear trends with age are expected for some LENA outcomes,^15,16^ we calculated *z* scores for AWC, PCCT, and CVC using age-specific LENA normative data.^16^ The *z* scores were calculated as follows: the difference between the observed 12-hour mean outcome (for AWC, PCCT, or CVC) and the corresponding 12-hour mean value for that outcome at that age according to normative LENA data was divided by the corresponding LENA normative standard deviation.^16^ The *z *score represents approximately how many standard deviations a given observation is from the normative mean value, for a specific age (in months).

Mixed-effects linear regression models were used to assess the effect of the intervention on AWC, PCCT, CVC, and Developmental Snapshot; a separate model was run for each outcome. A robust variance estimator was included, and clustering by individual was accounted for as a random effect; the model was fit via maximum likelihood. The unit of analysis was participant time point (time 1 or time 2), which represented intervention status. For each participant, time 1 (baseline) was a control observation, followed by an intervention follow-up observation at time 2. The time point was included as a binary variable to compare baseline and follow-up time points; additional covariates included child age, parent race and education, childcare during the recording (any vs none), and number of children in the home. We tested for interaction between the intervention and child age by including an interaction term in each model. We also assessed interaction between intervention and degree of participant engagement with the app (measured by number of clicks on the app links, received via text message) by adding to each model an interaction term between the number of clicks and intervention.

All hypothesis tests were 2-sided with an a priori significance level of *P* = .05. Stata statistical software version 14.2 (StataCorp) was used for all analyses.

## Results

**Figure 1.**
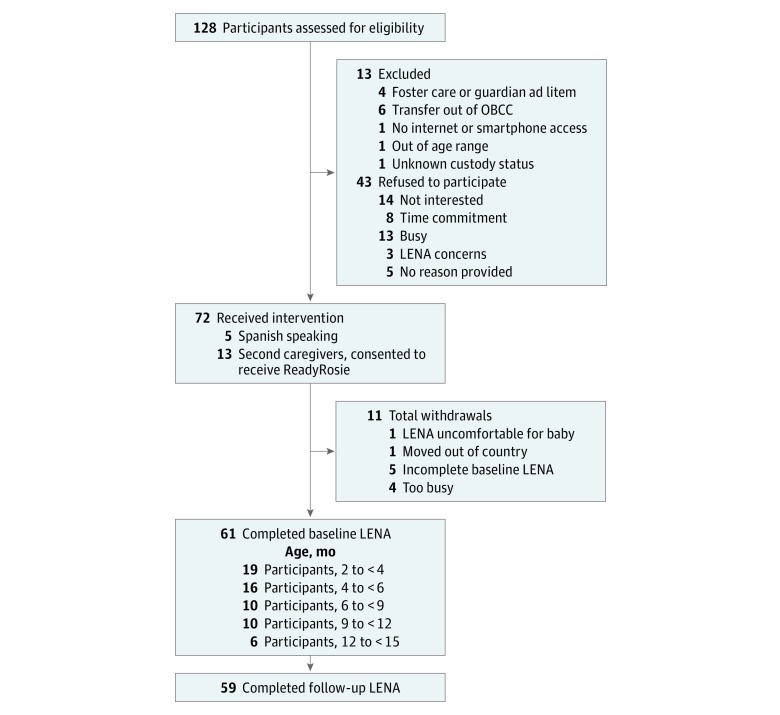
Cohort Enrollment Flowchart shows inclusion and exclusion criteria for cohort. LENA indicates Language Environment Analysis; OBCC, Odessa Brown Children’s Clinic.

We mailed 260 study invitation letters alerting families that we would attempt to contact them by phone for potential participation. In addition, 2 families contacted the study team after viewing a study recruitment flyer. We successfully contacted 128 families (49%); 13 (10%) were deemed ineligible, 43 (34%) declined, and 72 (64% of 115 eligible families) enrolled. A total of 11 families (15%) withdrew before completing the baseline LENA requirements. One family thought the LENA vest was uncomfortable for their baby to wear, 1 family moved out of the country, 4 families were too busy to complete the baseline assessment, and 5 did not complete the baseline assessment or provided only incomplete recordings (Figure 1).

A total of 61 families completed the baseline LENA, and 60 of those completed the baseline Developmental Snapshot; 59 (97%) of those families completed the follow-up LENA and the follow-up Developmental Snapshot. Among the 61 families, English was the primary language spoken in the home for 54 families (89%). The mean (SD) child’s age at baseline was 5.9 (3.3) months. The age ranges of the children at baseline were 2 to younger than 4 months (19 children), 4 to younger than 6 months (16 children), 6 to younger than 9 months (10 children), 9 to younger than 12 months (10 children), and 12 to younger than 15 months (6 children). The mean (SD) interval between time 1 and time 2 was 114.4 (27.9) days. There was no significant interaction between either intervention and baseline age or between interaction and number of app clicks (ie, the number of times parents clicked on links to view the videos) for any of the 4 models; thus, the interaction terms were not included in the final models.

Demographic data on participating families are summarized in Table 1. Overall, at time 2, the intervention was associated with significant improvements in *z *score for age for AWC (mean, 0.30; 95% CI, 0.05-0.55) and PCCT (mean, 0.29; 95% CI, 0.002-0.59) and for Developmental Snapshot score (mean, 6.59; 95% CI, 0.95-12.23), but there were no improvements in the *z* score for CVC (mean, −0.13; 95% CI, −0.49 to 0.24). Full results are presented in Table 2.

**Table 1.** Descriptive Characteristics of the Study Population at Baseline

Characteristic	Participants, No. (%) (N = 61)^a^
Child age at baseline Language Environment Analysis system measurement, mo	
2 to <4	19 (31)
4 to <6	16 (26)
6 to <9	10 (16)
9 to <12	10 (16)
12 to <15	6 (10)
Child’s race	
Asian	1 (2)
Black	6 (10)
White	29 (48)
Other (Native American or data missing)	2 (3)
>1 Race reported	22 (37)
Primary language spoken in child’s home	
English	54 (89)
Spanish	4 (7)
Other	3 (5)
Annual household income, $	
0-10 000	6 (10)
10 001-25 000	5 (8)
25 001-50 000	5 (8)
50 001-75 000	3 (5)
75 001-100 000	4 (7)
>100 000	36 (61)
Parents’ education	
High school diploma or general educational development test	11 (18)
2 y of college, associate degree, or professional training	6 (10)
4 y of college	16 (26)
2-3 y of postgraduate education	18 (30)
≥4 y of postgraduate education	10 (16)
Parents’ marital status	
Single, never married	12 (20)
Married, living together	45 (74)
Married, separated long term (>1 mo)	2 (3)
Other	2 (3)
Total children (aged <18 y) living in the home, No.	
1	30 (49)
2	23 (38)
3	6 (10)
4	2 (3)
Ever reported child being in childcare at any Language Environment Analysis time point	
No	55 (93)
Yes	4 (7)
Total clicks on ReadyRosie links (quartiles), No.	
0-16	13 (25)
17-37	14 (26)
38-58	13 (25)
59-200	13 (25)
Developmental Snapshot score, mean (SD)^b^	93.7 (18.4)

^a^ Percentages may not sum to 100% because of rounding. Numbers may not total 61 because of missing values, including race (1 missing), income (2 missing), number of clicks (8 missing), and childcare (2 missing).

^b^ The Developmental Snapshot score is a 52-item measure of parent-reported language skills for infants and toddlers.

**Table 2.** Word Count and Developmental Score Outcomes Associated With Talk It Up Intervention and Other Model Covariates

Model, Covariates	*z *Score, Mean (95% CI)^a^			Developmental Snapshot Score, Mean (95% CI)^b^
	Adult Word Count	Parent-Child Conversational Turn	Child Vocalization Count	
Adjusted model				
Participants, No.	57	57	57	57
LENA time point				
Baseline, preintervention	1 [Reference]	1 [Reference]	1 [Reference]	1 [Reference]
Follow-up, postintervention	0.30 (0.05 to 0.55)^c^	0.29 (0.002 to 0.59)^c^	−0.13 (−0.49 to 0.24)	6.59 (0.95 to 12.23)^c^
Age, mo				
2 to <6	1 [Reference]	1 [Reference]	1 [Reference]	1 [Reference]
6 to <9	−0.17 (−0.49 to 0.14)	−0.23 (−0.68 to 0.23)	−0.16 (−0.63 to 0.31)	1.05 (−5.65 to 7.76)
9 to <12	−0.42 (−0.76 to −0.10)^c^	−0.48 (−1.02 to 0.06)	−0.33 (−0.87 to 0.20)	3.22 (−3.11 to 9.55)
12 to <21	−0.48 (−0.95 to −0.02)^c^	−0.17 (−0.87 to 0.54)	0.23 (−0.53 to 1.00)	7.34 (−1.04 to 15.72)
Parents’ race				
White	1 [Reference]	1 [Reference]	1 [Reference]	1 [Reference]
Black	0.30 (−0.14 to 0.76)	0.37 (−0.16 to 0.90)	0.06 (−0.73 to 0.85)	8.01 (−1.08 to 17.09)
Other (Native American or data missing) or >1 race reported	−0.37 (−0.69 to −0.05)^c^	0.10 (−0.45 to 0.66)	−0.09 (−0.66 to 0.48)	2.10 (−7.24 to 11.44)
Parents’ education				
High school diploma or general educational development test	0.53 (−0.01 to 1.08)	0.77 (−0.01 to 1.54)	0.41 (−0.22 to 1.04)	−2.20 (−10.78 to 6.39)
2 y of college, associate degree, or professional training	−0.01 (−0.42 to 0.40)	0.05 (−0.53 to 0.63)	0.10 (−0.66 to 0.87)	−2.86 (−13.22 to 7.49)
4 y of college	1 [Reference]	1 [Reference]	1 [Reference]	1 [Reference]
2-3 y of postgraduate education	0.87 (0.50 to 1.26)^c^	0.82 (0.30 to 1.34)^c^	0.11 (−0.45 to 0.67)	−4.54 (−13.14 to 4.05)
≥4 y of postgraduate education	0.53 (0.15 to 0.91)^c^	0.40 (−0.11 to 0.90)	0.33 (−0.16 to 0.83)	−6.34 (−15.30 to 2.62)
Any childcare (vs none)	0.40 (−0.31 to 1.11)	0.49 (−0.40 to 1.37)	0.19 (−0.59 to 0.98)	−0.78 (−6.94 to 5.37)
No. of children in the home				
1	1 [Reference]	1 [Reference]	1 [Reference]	1 [Reference]
2	−0.62 (−0.87 to −0.37)^c^	−0.52 (−0.95 to −0.09)^c^	0.24 (−0.26 to 0.74)	−5.69 (−12.60 to 1.23)
3	−0.14 (−0.74 to 0.47)	−0.31 (−0.84 to 0.22)	0.23 (−0.39 to 0.85)	−7.05 (−14.48 to 0.38)
4	−0.86 (−1.40 to −0.33)^c^	−1.17 (−2.01 to −0.34)^c^	−0.70 (−1.32 to −0.08)	2.71 (−5.21 to 10.63)
Unadjusted model				
Participants, No.	61	61	61	60
LENA time point				
Baseline, preintervention	1 [Reference]	1 [Reference]	1 [Reference]	1 [Reference]
Follow-up, postintervention	0.18 (−0.06 to 0.41)	0.21 (−0.1 to 0.52)	−0.1 (−0.43 to 0.23)	7.76 (2.84-12.69)^c^

Abbreviation: LENA, Language Environment Analysis system.

^a^ The *z *score standardizes each observed value to the LENA normative values and represents the number of standard deviations of a given observation, from the (normative) mean value for a specific age (in months), according to normative LENA data. The *z *score is calculated as the difference between the 12-hour mean count (adult word count, parent-child conversational turns, or child vocalization count) and the normative LENA 12-hour mean value for that count at that age divided by the LENA normative standard deviation.

^b^ The Developmental Snapshot score is a 52-item measure of parent-reported language skills for infants and toddlers measured at each time point.

^c^ *P* < .05.

## Discussion

We found that a combination of feedback, coaching, and links to age-based videos of parent-child models of interaction was associated with a significant difference in AWC, PCCTs, and Developmental Snapshot score after the intervention. The strength of association between the intervention and outcomes of interest by conventional norms would be viewed as moderate for AWC and PCCT and large for Developmental Snapshot score. Given recent data^17^ showing that the early language environment is associated with both short- and long-term benefits, these findings, if confirmed and extended, are potentially important.

Promotion of parent-child interactions has long been a goal of WCVs in primary care. What have been lacking are practical strategies to help parents implement the recommendations. Our finding that, at least in the short term, actively disseminated video examples are associated with some benefit provides a potential model for a scalable intervention. Future larger studies should explore ways of sustaining or improving the association seen in the short term.

### Limitations

These findings should be viewed in light of some important limitations. First, our sample size is small and derived from a single pediatric clinic. The extent to which these findings could be generalized to other locales is unknown. Second, we did not use an experimental design. However, we used age-based normalized outcomes with *z *scores for age, so that changes in outcome we see over time are not purely those expected as the result of normal child development. In addition, children ranged in age from 2 to 12 months at enrollment and also varied somewhat at follow-up; by taking into account both age and intervention status, our mixed-effects model is able to compare, for example, a child who was 9 months old at baseline with a child who was 9 months old at the second intervention. Third, although our sample was drawn from an urban clinic, it was skewed toward a higher socioeconomic status population. Whether similar effect sizes could be achieved in exclusively lower-income populations is unclear. Our sample was not of sufficient size to meaningfully stratify by income. Fourth, this study, like most behavioral interventions, was not blinded. This limitation would not affect the LENA data, which are objectively collected, but could affect the Developmental Snapshot score. Fifth, it is unclear which aspects of the intervention drove the observed effects. As such, it is impossible to know whether the app would be effective alone or whether coaching is also needed. Future studies could explore this question.

## Conclusions

The Talk It Up intervention, including a smartphone app and coaching, holds promise for improving the linguistic environment of early childhood and, notably, has important implications for using technology as part of the solution. Future, larger studies are warranted.
